# Upregulation of miR-216a-5p by Lentinan Targeted Inhibition of JAK2/STAT3 Signaling Pathway to Reduce Lung Adenocarcinoma Cell Stemness, Promote Apoptosis, and Slow Down the Lung Adenocarcinoma Mechanisms

**DOI:** 10.3389/fonc.2021.778096

**Published:** 2021-11-25

**Authors:** Quan Chen, Yiming Zheng, Xia Chen, Pengfei Ge, Pengcheng Wang, Bingbing Wu

**Affiliations:** Department of Thoracic Surgery, Hospital Affiliated 5 to Nantong University (Taizhou People’s Hospital), Taizhou, China

**Keywords:** lung adenocarcinoma (LUAD), cell stemness, lentinan, miR-216a-5p, JAK2/STAT3 signaling pathway

## Abstract

To investigate the effect of Lentinan (LNT) on lung adenocarcinoma (LUAD) cell stemness and its mechanism. In this study, we founded that LNT significantly reduce the cell proliferation, activity, migration, invasion, and stemness of LUAD cells, and promote their apoptosis compared with the control group *in vitro*. Moreover, LNT significantly inhibited the volume and weight of tumors of nude mice *in vivo*. At the same time, LNT can significantly up-regulate miR-216a-5p levels and reduce the protein expression of phospho-JAK2 (Y1007/1008) and phospho-STAT3 (Tyr705), thereby inhibiting the JAK2/STAT3 signaling pathway. Interfering with miR-216a-5p expression and activating the JAK2/STAT3 signaling pathway can significantly reverse LNT inhibitory effects on LUAD. Collectively, LNT can inhibit the JAK2/STAT3 signaling pathway by up-regulating miR-216a-5p, reducing stemness, and promoting LUAD cells apoptosis, then slow down LUAD occurrence and development, providing concepts and experimental foundation treating patients with LUAD.

## Introduction

Lung cancer is a malignant tumor with peak morbidity and mortality rates among various cancers globally (1). In clinical practice, multidisciplinary treatment with surgery, chemotherapy, and radiotherapy is its primary treatment. Though the patient survival time has been perpetuated after treatment, results are generally poor. Most patients will still die of cancer relapse or metastasis, leading to an around 15% 5-year survival rate (2). Thus, the invasion and metastasis mechanisms of lung cancer have been a research hot spot in oncology. In 1994, Lapidot et al. first identified and isolated cancer stem cells (CSCs) from acute myeloid leukemia patients (3). As studies deepen, CSCs have been isolated and identified from different tumor tissues, including breast cancer (4), pancreatic cancer (5), and lung cancer (6). Studies have confirmed that a significant increase in CSCs was also found in primary malignant cells in the ascites of patients with high-grade serous ovarian cancer (7). CSCs are subpopulations of tumor cells with self-renewal, differentiation, and homeostatic control capabilities like normal tissue stem cells. Studies have found that CSCs have stronger radio-resistance than typical tumor cells and are considered to be radio-resistant in tumor tissues, which is the key to tumor migration and invasion (8). Recent studies have revealed that CSCs are critical in tumor occurrence and development. Compared with adult tumor cells, CSCs possess a stronger ability to repair DNA damage and scavenge oxygen free radicals (9). It has been confirmed that the oncogenic ability *in vivo* and invasion and metastasis ability of CSCs are also significantly higher than those of adult tumor cells (10). The above characteristics of CSCs allow them to initiate tumor development *in situ* or migrate distally, leading to tumor migration and invasion. At present, there are also drugs for the treatment of tumor cell stemness in the clinic., such as Napabucasin, and it has the effect of inhibiting self-renewal and inducing apoptosis in colorectal cancer, pancreatic cancer, non-small cell lung cancer, and prostate cancer stem cells (11–14). But it can cause adverse reactions in patients (15). Thus, medical research has hot spots to elaborate the pathogenesis of stemness induction in lung cancer and investigate and develop effective preventive and therapeutic drugs.

Traditional Chinese medicine has been widely used in Asia for thousands of years. Chinese herbal medicines and proprietary Chinese medicines have become alternative treatments for a large number of patients with cancer because of their good efficacy (16). Lentinan (LNT) is a plant-derived natural polysaccharide (Lentinus edodes), which is broadly regarded as a complementary drug supplement that has noteworthy anti-cancer, anti-viral, anti-inflammatory, immunomodulatory, anti-coagulant, and anti-tumor effects (17). Related studies have shown that LNT has strong anti-tumor activity (17). Research has revealed that LNT is able to effectively inhibit cell proliferation, differentiation, growth, and senescence (18) and effectively prevent cancer development triggered *via* chemical or viral carcinogens (19). Clinically, LNT can boost chemotherapy effects and increase patient survival rates of gastric, colon, breast, and lung cancer (20). However, there are limited reports on the impact of LNT on lung adenocarcinoma (LUAD), the role of LUAD stem cells, and related mechanisms.

Endogenous small non-coding RNAs or microRNAs (miRNAs) are 19 to 25 nucleotides in length, encoded by higher eukaryotic genomes (21). In recent years, many reports have presented dysregulated miRNAs having a close relationship with tumor occurrence and development and may contribute as oncogenes or tumor-suppressive genes (22). It has been found that miR-216a is critical in a range of malignancies. For instance, it has been shown that miR-216a inhibits pancreatic cancer cell growth and promotes apoptosis by acting on the JAK2 gene (23), and prevents glioma cell proliferation and invasion by aiming for and regulating protein kinase Cα (24). Also, gene chip analysis of 104 pairs of lung cancer tissues and tissues by Yanaihara et al. showed that miR-216a was also down-regulated (25). Based on the above studies, we hypothesize that inhibitory effects can be brought on lung cancer stem cells by miR-216a-5p. However, there is no research report on the role of miR-216a-5p in LNT affecting lung cancer stem cells.

It has been shown that the Janus kinase2/signal transducer and activator of the transcription3 (JAK2/STAT3) signaling pathway have been revealed to be a vital part of the progression of lung cancer. Blocking the JAK2/STAT3 signaling pathway in non-small cell carcinoma (NSCLC) inhibits the proliferation, angiogenesis, invasion, and migration ability of NSCLC and additionally inhibits the development of lung cancer (26, 27). Meanwhile, it has been discovered that CSCs growth regulation involved the JAK2/STAT3 signaling pathway in recent years. Marotta et al. (28) confirmed that the JAK2/STAT3 is compulsory for breast cancer stem cell growth. Additional research revealed that breast cancer stem cells stemness characteristics could be lost by JAK2/STAT3 signaling pathway blockage (29). It has been documented that miR-216a-5p can inhibit epithelial-mesenchymal transition (EMT) in gastric cancer migration and invasion by targeted inhibition of the JAK2/STAT3 signaling pathway (30). Therefore, in this study, clinical cases and *in vitro* cell experiments were used to study the effects of lentinan on the proliferation, migration, invasion, apoptosis, and cell stemness of lung adenocarcinoma cells, as well as the involvement of miR-216a-5p and JAK2/STAT3 signaling pathways. This study investigated this speculation and provided new ideas for LUAD treatment.

## Materials and Methods

### Patient Tissue Samples

Tumor tissues and adjacent normal tissues of diagnosed LUAD patients in our hospital from January 2016 to June 2018 were collected. The patients’ basic information was recorded, including age, gender, blood pressure, smoking, alcoholism, tumor staging, distant metastasis, and lymphatic metastasis. All patients were confirmed by three pathologists for postoperative pathological staging and did not receive chemotherapy or radiotherapy. They had no history of major systemic diseases with a confirmation of primary lesion by pathological examination. Every patient gave informed and signed the consent. This study was approved by the Ethics Committee of Hospital Affiliated 5 to Nantong University (Taizhou People's Hospital) (Number: KY 202003401).

### Cell Culture and Treatment

Human LUAD cell lines H1299 and H460 were bought from the Cell Bank of Shanghai Institute of Biochemistry and Cell Biology, Chinese Academy of Sciences and cultured in the RPMI-1640 Medium (GIBCO, USA), including 10% fetal bovine serum (FBS), 100 U/ml penicillin, and 100 μg/ml streptomycin. All cells were maintained in an incubator of 5% CO_2_ at 37°C.

### Cell Transfection

A 6-well plate was used to seed H1299 and H460 cells with a 5×10^5^ cells/well density. When fused to a density of 60% to 70%, the miR-216a-5p inhibitor (in-miR-216a-5p) was transfected with Lipofectamine 2000 kit (Thermo Fisher, USA) protocol according to the manufacture’s instruction. After 6 h of incubation, cells were cultured again for 48 h after replacing with a new medium.

### CCK8 Assay

Trypsin was used to digest H1299 and H460 cells, seeded in a 96-well culture plate at 5000 cells/well density, and cultured for 24 h for wall adhesion. After treating the cells with assorted Lentinan (0 μM, 5 μM, 10 μM, 50 μM, 100 μM, 200 μM) concentrations of for 24 h, 10 μL of 5 g/L CCK8 solutions was inserted into individual wells, and incubate for 4 h. Afterward, the supernatant was removed, and the light absorption value was measured at 450 nm wavelength on a fluorescence enzyme-linked analyzer. Experimentation was completed in triplicate.

### Quantitative Real-Time PCR (qRT-PCR)

The TRizol method (Gibco, USA) was utilized to extract total RNA from lysed cells or tissues. The PrimeScript TMRT reagent Kit protocol was followed for performing reverse transcription. qRT-PCR kit instructions (Japan, TaKaRa) was followed and performed to obtained cDNA with 42°C/30 min and 85°C/15 s reaction conditions. The reaction system was: pre-denaturation at 95°C for 20 s; 95°C for 10 s for denaturation, 55°C for 20 s extension, 70°C for 20 s for annealing, and repeated the process 40 times. The 2 ^− ΔΔCt^ method was applied to calculate relative miRNA expression with an internal reference of U6. Primer sequences are presented in **Table 1**.

**Table 1:** Primer sequence.

Primer	Primer Sequence
miR-216a-5p	F : 5′-ACATCCTCGGCCAGTAAGACTG-3′
	R : 5′-GTCGACCAGATTGCGTTCG-3′
U6	F : 5′-CTCGCTTCGGCAGCACA-3′
	R : 5′-AACGCTTCACGAATTTGCGT-3′

### Cell Colony Formation Assay

0.25% trypsin was used to digest cells in each group and centrifuged at 1000 r/min for 3 min before discarding the supernatant. After 3 times washing with sterile PBS solution, a single-cell suspension was prepared with the addition of a culture medium. The H1299 and H460 cells concentrations were altered to 1000 cells/ml and evenly planted and cultured in 6-well plates sterile. Culture medium was replaced every 3 d. Colonies were visible to the naked eye after 14 d. After termination of culture, 0.1% crystal violet solution was used to stain the cells and photographed *via* an inverted microscope, and the number of colonies was counted. Experimentation was completed in triplicate.

### Cell Apoptosis

Logarithmic growth phase cells from each group were taken and digested with trypsin before centrifugation at 3,000 r/min for 10 min at 4°C. The supernatant was removed. In correspondence to the Annexin V-FITC/PI Cell Apoptosis Detection Kit (Southern Biotech, USA) instructions. 5 μl Annexin V-FITC and 10 μl PI were combined, respectively. The cells were gently mixed and incubated at room temperature for 15 min away from light exposure to detect apoptosis using a FACSCalibur Flow Cytometry (BD, USA).

### Stem Cell Sphere-Forming Assay

Ultra-low adsorption 6-well cell culture plates (Corning, USA) were used to seed cells in each group at a density of 1 × 10^3^ cells/well, 3 duplicate wells were set for each concentration, suspended in serum-free DMEM/F12 (1:1) culture medium containing stimulating factors such as B27, EGF, bFGF, heparin, and insulin, placed in a 5% CO_2_ cell incubator at 37°C. Stimulating factors were added every 3 d. After 14 d, cell growth and sphere-formation were observed under an inverted microscope, counted, and the mean value determined.

### Caspase3 Activity Assay

96-well plates were used to seed cells at 2 × 10^8^/L density. After 48-hour treatment, cells were cleaned by precooled PBS before the addition of 25 μL cell lysate, allowing them to stand for 5 min on ice, and then 200 μL of Ac-DEVD-AMC (caspase-3 tetrapeptide fluorescent substrate, abcom, USA) was added. After 1-hour incubation at 37°C in the dark, the fluorescence intensity was assessed on a multifunctional microplate reader with a 380 nm excitation wavelength and 420 nm emission wavelength. The units were expressed as relative fluorescence units (RFU).

### Cell Invasion

2.5 × 10^4^ cells were resuspended in 100 μl serum-free 1640 medium, added to the Transwell plate upper chamber, and coated with Matrigel (Corning, USA). Then 500 μl of 1640 containing 20% FBS was inserted into the lower chamber. Following culture for 24 hours, upper chamber unpermeated cells were gently wiped using a cotton swab. The cells were removed from the chamber, fixed with methanol for 15 min, and stained with crystal violet solution. A microscope was used to examine cells with five fields randomly chosen per sample used for counting.

### Cell Migration

2.5 × 10^4^ cells were resuspended in 100 μl of 1640 medium without FBS and planted in a Transwell chamber. 500 μl of 1640, including 20% FBS, was added to the lower chamber. Following culturing for 24 h, a cotton swab was used to gently wipe the impermeable cells in the upper chamber. The chamber was taken out, fixed for 15 min with methanol, and stained with crystal violet solution. A microscope was used to examine cells with five fields randomly chosen per sample used for counting.

### Western Blot

Total proteins was extracted using RIPA lysate (Gibco, USA) from cells and tumor tissues. The total protein concentration was determined by BCA Kit (Beyotime Biotechnology, China). After the separation with SDS-PAGE electrophoresis, proteins were transported to PVDF membranes and then blocked for 2 h using TBST blocking buffer comprising of 5% skimmed milk. The membranes were incubated with primary antibody rabbit polyclonal antibody CD90 (ab92574, Abcam, UK), mouse monoclonal antibody CD133 (ab264538, Abcam, UK), rabbit monoclonal antibody JAK2 (ab108596, Abcam, UK), rabbit monoclonal antibody p-JAK2 (ab32101, Abcam, UK), rabbit monoclonal antibody STAT3 (ab68153, Abcam, UK), rabbit monoclonal antibody p-STAT3 (ab76315, Abcam, UK) at a dilution of 1:1000 overnight at 4°C. The following day horseradish peroxidase (HRP)-labeled secondary antibody was added, incubated for 2 h, developed with chemiluminescence, and placed in a gel imaging system to collect images analyzing by Image J software.

### Xenograft Nude Mouse Model

Twelve male BALB/c nude mice (age, 6-8 weeks, body weight, 20-25 g) were purchased from Laboratory Animal Services Centre, Nantong University. All mice were housed in the specific pathogen-free (SPF) condition. 0.25% trypsin was used to digest logarithmic growth phase H1299 cells and H1299 cells treated with 100 μM LNT to set up a 5 × 10^7^/mL single-cell suspension 200 µl was subcutaneously injected into the anterior axilla of mice in each group. Food and water were provided ad libitum during the experiment. Tumor length and width were measured every 4 days *via* a vernier caliper. Tumor volume = (length × width^2^)/2 formula was used to calculate tumor volume. Mice were euthanized, the tumor tissues of each group were weighed and collected 28 days later.

### Immunohistochemistry (IHC)

Nude mice tumor tissues were taken and fixed in 10% formaldehyde for paraffin-embedded sectioning (5 μM). The performance of IHC was followed by the immunohistochemical kit’s (Invitrogen, USA) instructions. Sections were incubated with primary antibody Ki-67 (ab15580, Abcam, UK) overnight at 4°C. Following subsequent incubation with lgG-HRP, color was developed with diaminobenzidine (DAB) chromogen solution. The staining of cells was observed under a light microscope.

### Statistical Analysis

Statistical analysis of experimental data was completed with the use of SPSS 22.0 software and stated as mean ± standard deviation (SD), t-test was applied for comparing the differences between two groups, and differences between multiple groups was compared with the one-way analysis of variance. Experimentation was completed in triplicate with P < 0.05 representing statistical significance.

## Results

### LNT Can Inhibit Human Lung Adenocarcinoma Cell Malignant Development

**Figure 1:**
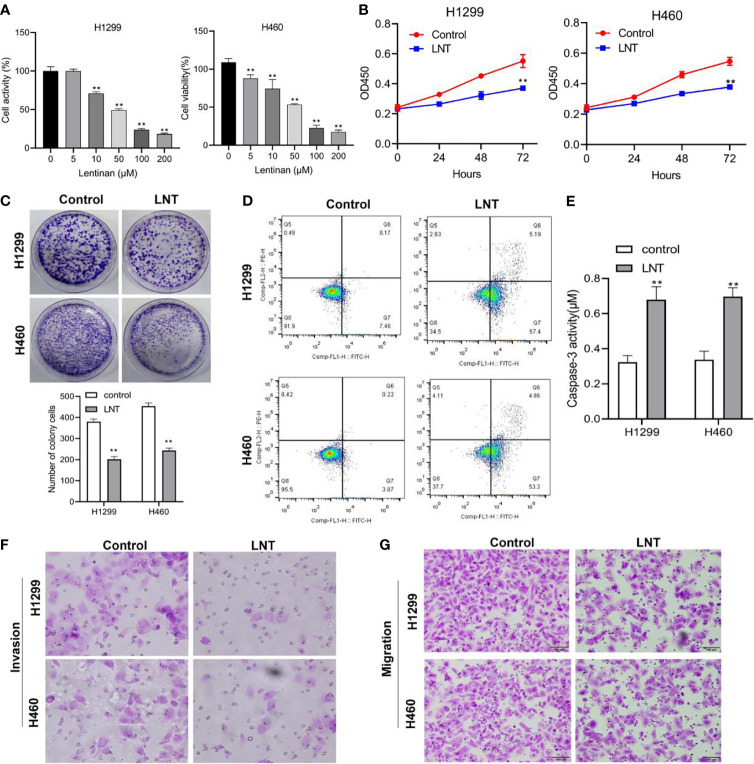
LNT effects on human lung adenocarcinoma cell malignant development. **(A)** CCK-8 detects H1299 and H46 cell viability under the intervention of different concentrations of LNT; After treatment of H1299 and H460 cells with 100 μM of LNT, **(B)** CCK-8 assay was used to detect cell proliferation at 24, 48, 72 h; **(C)** The proliferation capacity of cells was determined by colony assay; **(D)** Cell apoptosis was measured *via* flow cytometry; **(E)** Caspase3 activity was examined; **(F, G)**. Transwell assay were performed to detect cell invasion **(F)** and migration **(G)**. All experiments were repeated three times. **P < 0.01 *vs*. control group.

The malignant development of LNTon LUAD cells was first investigated by CCK-8 to detect H1299 and H460 cell viability at various concentrations of LNT. The results showed that the H1299 and H460 cells viability was significantly decreased in the LNT group (10, 50, 100 and 200 μM) compared to the Control group, and the change rate of cell viability was largest at 100 μM (**Figure 1A**). Additionally, through CCK-8, it was discovered that 100 μM LNT treated H1299 and H460 cell proliferation ability was lesser when compared with the control group (**Figure 1B**). The colony formation experiment also proved that LNT could inhibit LUAD cell proliferation capacity (**Figure 1C**). Further apoptosis experiments found that LNT can promote LUAD cell apoptosis and increase Caspase-3 activity (**Figures 1D, E**). In addition, LNT can reduce LUAD cell H1299 and H460 invasion and migration abilities (**Figures 1F, G**). This shows that LNT can inhibit LUAD cell malignant development and promote cell apoptosis.

### LNT Inhibits the Stemness of Lung Adenocarcinoma Cells

**Figure 2:**
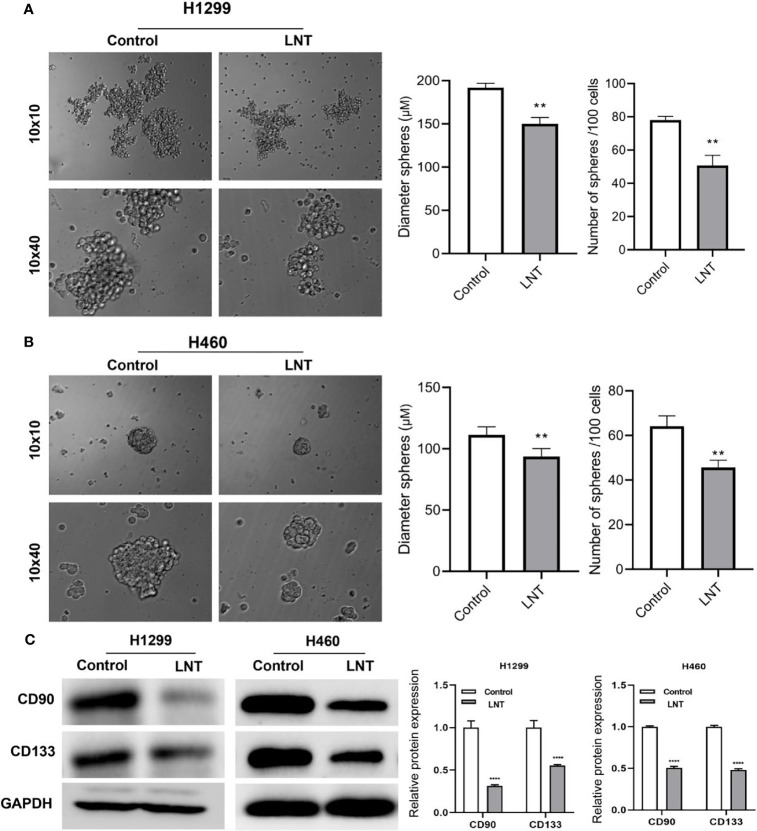
LNT can inhibit the stemness of lung adenocarcinoma cells. After treatment of H1299 and H460 cells with 100 μM of LNT, **(A)** Stem cell sphere-forming assay to detect the sphere-forming efficiency of H1299 cells; **(B)** Stem cell sphere-forming assay to detect the sphere-forming efficiency of H460 cells; **(C)** Stem cell marker proteins CD90 and CD133 expression levels detected by Western blot. **P < 0.01 and ****P < 0.0001 *vs*. control group.

Stem cell sphere-forming assay was further used to identify the sphere-forming efficiency of LUAD cells, the diameter of cell pellets, and the number of cell pellet formations. In contrast, cell surface stem cell markers CD90 and CD133 expression levels were detected using Western Blot. The findings revealed cell sphere-forming efficiency in the LNT group (**Figures 2A, B**), and CD90 and CD133 expression (**Figure 2C**) was radically decreased when in comparison with those in the control group. This result suggested inhibition of stemness of LUAD cells by LNT.

### LNT Can Up-Regulate miR-216a-5p and Inhibit the JAK2/STAT3 Signaling Pathway

**Figure 3:**
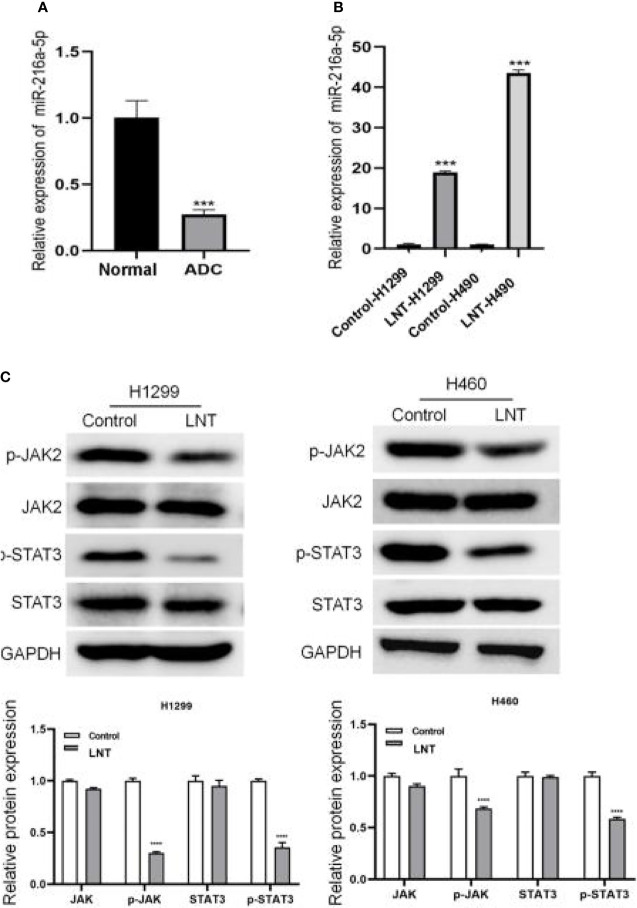
LNT effects on miR-216a-5p and JAK2/STAT3 signaling pathway. **(A)** miR-216a-5p expression in human lung adenocarcinoma tissues and adjacent normal tissues by qRT-PCR; Per group n = 15. **(B)** qRT-PCR was used to measure the miR-216a-5p expression in H1299 and H460 cell; **(C)** Western blot was performed to detect JAK2/STAT3 signaling pathway related protein expression in H1299 and H460 cells. ***P < 0.001 and ****P < 0.0001*vs*. control group.

Further, we explored the molecular mechanism by which LNT inhibits LUAD cell function. The results demonstrated a lowly expressed miR-216a-5p in LUAD tissues (**Figure 3A**). It was further found that LNT could significantly up-regulate the expression of miR-216a-5p in H1299 and H460 cells (**Figure 3B**). At the same time, LNT inhibited protein expression of phospho-JAK2 (Y1007/1008) and phospho-STAT3 (Tyr705) in LUAD cells and significantly reduced the p-JAK2/JAK2 and p-STAT3/STAT3 ratios (**Figure 3C**). This result suggested that LNT could up-regulate miR-216a-5p and inhibit JAK2/STAT3 signaling pathway activation.

### Low miR-216a-5p Expression or IL-6 Can Inhibit the Tumor Suppressor Effect of LNT on Lung Adenocarcinoma Cells and Promote Cancer Cell Stemness

**Figure 4:**
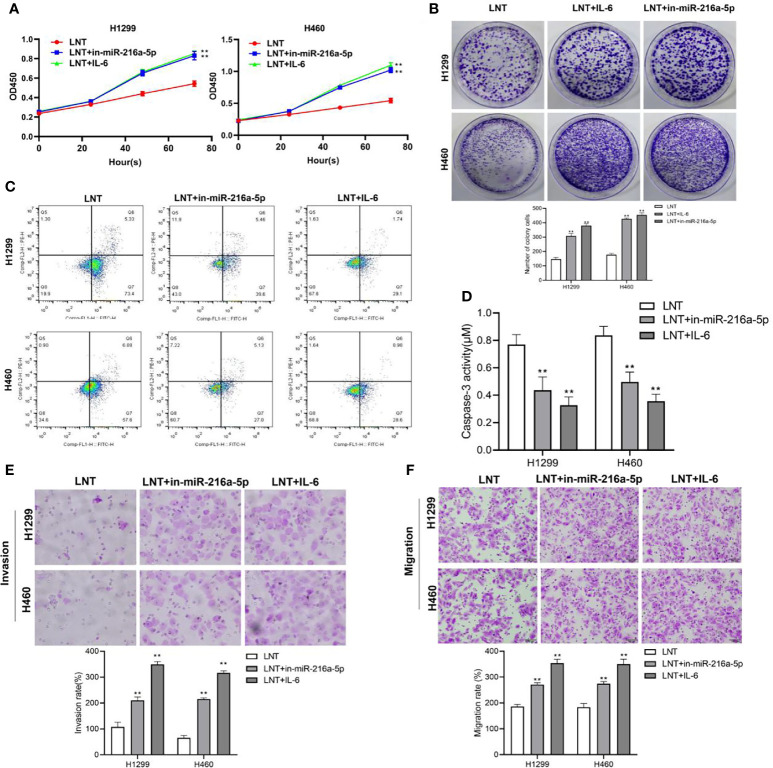
Interference with miR-216a-5p or the addition of IL-6 on LNT anti-cancer effects. **(A)** CCK8 detects H1299 and H460 cell proliferation in each group; **(B)** Cell colony experiment detects H1299 and H460 cell viability in each group; **(C)** H1299 and H460 cell apoptosis in each group detected by flow cytometry; **(D)** Detection of H1299 and H460 cell Caspase3 activity in each group; **(E)** Transwell detects H1299 and H460 cell invasion ability in each group; **(F)** Transwell detects of H1299 and H460 cell migration ability in each group. **P < 0.01 *vs*. LNT group.

We interfered with the miR-216a-5p and JAK2/STAT3 signaling pathway in LNT affecting LUAD development; we interfered with the miR-216a-5p expression and treated the cells with the JAK2/STAT3 pathway activator IL-6. The results displayed that in comparison with the LNT group, miR-216a-5p interference or IL-6 addition could significantly promote LUAD cell proliferation and cell viability and increase cell invasion and migration capabilities while inhibiting cell apoptosis and Caspase-3 Activity (**Figures 4A–F**).

**Figure 5:**
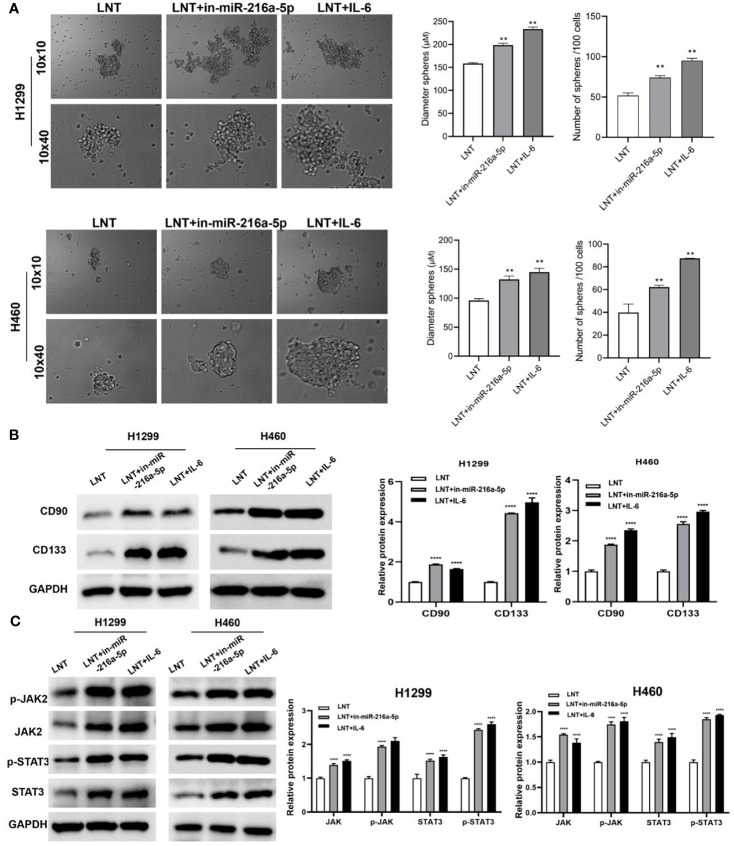
The effect of interfering with miR-216a-5p or IL-6 on LNT promoting cancer cell stemness and the JAK2/STAT3 pathway. **(A)** Stem cell sphere-forming assay to detect the spheroidization efficiency of H1299 and H460 cells in each group; **(B)** Western blot to detect H1299 and H460 cells stem cell marker proteins CD90 and CD133 expression; **(C)** Western blot to detect H1299 and H460 cells JAK2/STAT3 signaling pathway protein expression in each group. **P < 0.01 and ****P < 0.0001 *vs*. LNT group.

In addition, inhibiting miR-216a-5p and activating the JAK2/STAT3 pathway can promote tumor cell spheroidization, up-regulating the levels of stem marker proteins CD90 and CD133 (**Figures 5A, B**). Simultaneously, the expression of phospho-JAK2 (Y1007/1008) and phospho-STAT3 (Tyr705), and the ration of p-JAK2/JAK2 and p-STAT3/STAT3 were also significantly increased (**Figure 5C**). This result suggests that interfering with miR-216a-5p or IL-6 expression can reverse the tumor suppressor effect of LNT on LUAD cells and promote cancer cell stemness.

### LNT Can Inhibit the Growth of Lung Adenocarcinoma *In Vivo*

**Figure 6:**
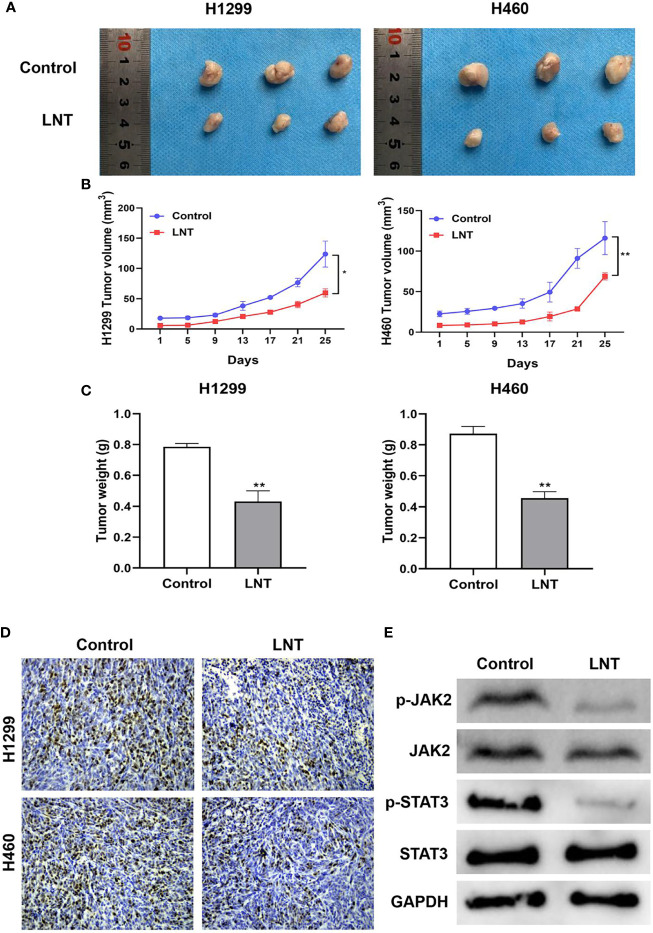
LNT effects on the growth of lung adenocarcinoma *in vivo*. **(A)** Tumors of nude mice in each group after inoculating H1299 and H460 cells with tumor; **(B)** Tumor size of nude mice in individual groups after inoculating H1299 and H460 cells with tumor; **(C)** Tumor weight of nude mice in individual groups after tumor inoculation in H1299 and H460 cells; **(D)** IHC used to detect Ki-67 expression in tumor tissues of nude mice in individual groups; **(E)** JAK2/STAT3 signaling pathway related proteins expression in tumor tissues detected using Western blot. Per group n = 6. *P < 0.05 and **P < 0.01 *vs*. control group.

Finally, the inhibitory effect of LNT on LUAD was further verified by the animal *in vivo* experiments. An *in vivo* model of LUAD was constructed in nude mice. The outcomes proved that the LNT group’s tumor volume, weight, and Ki-67 expression levels were considerably lower than the control group (**Figures 6A–D**). In addition, the protein expression levels of p-JAK2 (Y1007/1008) and p-STAT3 (Tyr705) in the tumor tissues of nude mice in the LNT group were significantly reduced by western blot (**Figure 6E**). This study indicates that LNT can inhibit LUAD growth by inhibiting the JAK2/STAT3 signaling pathway *in vivo*.

## Discussion

Shiitake mushroom has a long history as a medicine in China. Lentinan is an integral derivative of the Shiitake mushroom, which is primarily comprised of β-glucan that has anti-tumor, anti-inflammatory, and anti-diabetic properties. It was approved to be used as a gastric cancer drug in Japan in 1985 (31). In addition, Lentinan can activate TXNIP-NLRP3 inflammasomes *via* the ASK1/p38 MAPK signaling pathway and cooperate with paclitaxel to treat non-small cell lung cancer (32). Simultaneously, Lentinan can activate the NRF2-ARE signaling pathway to significantly avoid renal injury provoked by the chemotherapy drug cisplatin (33). Lentinan is widely used clinically in China and Japan to treat cancer and can be taken orally or intravenously. However, its effect on LUAD is still unclear, and its mechanism of action on the stemness of LUAD cells has yet to be thoroughly studied. According to findings in this paper, human LUAD cell lines H1299 and H460 were employed to explore the anti-tumor mechanism of Lentinan. The findings revealed that the LNT group’s cell viability, invasion, and migration ability were significantly decreased compared to the control group. In contrast, the apoptosis ability was increased considerably, indicating that LNT could significantly inhibit the biological activity of LUAD cells and slow down the occurrence and development of LUAD, which was consistent with the above literature reports.

Lung CSCs are believed to be one of the factors for poor prognoses, such as drug resistance, metastasis, and recurrence in patients’ treatment (34), so elucidating the source or generation mechanism of lung cancer CSCs is vital to investigate effective tumor therapeutic targets. CD90 and CD133 were first discovered as important marker molecules of hematopoietic stem cells (35, 36). It is also one of the surface marker molecules of adult stem cells such as prostate cancer stem cells (37). CD90 and CD133 have been demonstrated to possess the traits of CSCs in lung cancer cells, including clonosphere growth in serum-free medium containing EGF, rapid *in vivo* tumorigenicity (38), and resistance to chemoradiotherapy (39). Therefore, they both have been employed as molecular markers for CSC isolation and identification of lung cancer CSC (40). Western blot was applied in this article to detect CD90 and CD133 expression levels in every group of cells. It was found that LNT can significantly reduce CD90 and CD133 expression in LUAD cells, suggesting that LNT has the biological function of reducing cell stemness.

After determining the inhibitory effect of LNT on the proliferation, invasion, migration, and cell stemness of LUAD, elaborating the particular mechanism leading to this effect has turned into our next research focus. miRNA can sustain cell homeostasis through negative gene regulation, and its abnormal expression is related to numerous cancers by behaving as a tumor suppressor and carcinogens (41). miRNA plays a part in nearly all characteristics of cancer biology, including proliferation, apoptosis, invasion/metastasis, and angiogenesis, and therefore is indispensable in tumor diagnosis and treatment. It has been stated that miR-216a-5p can inhibit the occurrence of pancreatic cancer by targeting TPT1/mTORC1 (42) and also inhibit the malignant progression of small cell lung cancer (43). Yet, its effect on LUAD and its mechanism is still unclear. A significant number of research reports have established that the JAK2/STAT3 signaling pathway is the most critical downstream signaling pathway for growth and development. Intracellular JAK2 kinase is externally activated and phosphorylates tyrosine at position 705 (Y705) at the carboxy-terminus of STAT3 in the cytoplasm. Phosphorylated STAT3 monomers enter the nucleus by interacting with tyrosine residues phosphorylated by another STAT3 molecule at position 608 of the SH2 domain to form dimers that bind to their specific DNA response elements and regulate target gene transcription, exerting different biological effects, including the regulation of physiological functions such as cell growth, apoptosis, invasion, migration, and angiogenesis (44). Lee et al. (45) reported that Brustatol could inhibit EMT by suppressing p-STAT3 (Y705) expression, resulting in anti-HCC metastasis. Yang et al. (46) stated that tumor-associated macrophages could activate STAT3 in tumor cells *via* the paracrine pathway, followed by up-regulation of SOX2 expression, and breast cancer cells acquire a stemness phenotype. Kryczek et al. (47) proved that inflammatory cells in the colorectal cancer microenvironment could stimulate stemness transition in tumor cells by secreting IL-22 and activating STAT3 in tumor cells. Studies have shown that miR-216a-5 can regulate the JAK2-STAT3 axis and affect the proliferation and apoptosis of osteoarthritis chondrocytes (48). This study found that LNT caused a significant increase in miR-216a-5p expression levels in tumor cells; decreased related protein expression in the JAK2/STAT3 pathway. At the same time, inhibition of miR-216a-5p or IL-6 induced cells after tumor cell stemness transformation is promoted, and the effect of LNT is reversed, indicating that LNT is involved in cell stemming by regulating miR-216a-5p targeting and mediating the JAK2/STAT3 signaling pathway. Therefore, based on the outcomes gained, it is speculated that LNT can upregulate miR-216a-5p, restrain the JAK2/STAT3 signaling pathway, promote apoptosis, reduce the stemness of l LUAD cells, and slow down the occurrence and development of LUAD.

## Conclusion

In summary, Lentinan can up-regulate miR-216a-5p and inhibit the JAK2/STAT3 signaling pathway expression, thereby significantly reducing the proliferation, migration, invasion, and cell stemness of LUAD cell lines H1299 and H460 and promote cell apoptosis. The conclusions in this study also need to be verified by subsequent clinical tissue samples, which are worthy of systematic in-depth study to provide the experimental basis for malignant LUAD tumor treatment.

## Data Availability Statement

The original contributions presented in the study are included in the article/supplementary material. Further inquiries can be directed to the corresponding author.

## Author Contributions

QC: conceptualization, method design, data analysis and sorting, writing - original draft, and writing - review and editing. YZ: writing - review and editing. XC: data analysis and sorting. PG: data analysis and sorting and writing - original draft. PW: writing - review and editing. BW: conceptualization, method design, and writing - review and editing. All authors contributed to the article and approved the submitted version.

## Funding

This work was funded by Nantong University Clinical Medicine Special Project Fund (Grant No. 2019JZ008).

## Conflict of Interest

The authors declare that the research was conducted in the absence of any commercial or financial relationships that could be construed as a potential conflict of interest.

## Publisher’s Note

All claims expressed in this article are solely those of the authors and do not necessarily represent those of their affiliated organizations, or those of the publisher, the editors and the reviewers. Any product that may be evaluated in this article, or claim that may be made by its manufacturer, is not guaranteed or endorsed by the publisher.
